# Early on-treatment C-reactive protein and its kinetics predict survival and response in recurrent and/or metastatic head and neck cancer patients receiving first-line pembrolizumab

**DOI:** 10.1007/s10637-023-01388-x

**Published:** 2023-08-21

**Authors:** Markus Haas, Alexander Lein, Thorsten Fuereder, Julia Schnoell, Faris F. Brkic, David T. Liu, Lorenz Kadletz-Wanke, Gregor Heiduschka, Bernhard J. Jank

**Affiliations:** 1 Department of Otorhinolaryngology, Head and Neck Surgery, Medical University of Vienna, Waehringer Guertel 18-20, Vienna, 1090 Austria; 2 Division of Oncology, Department of Medicine I, Medical University of Vienna, Waehringer Guertel 18-20, Vienna, 1090 Austria

**Keywords:** Immunotherapy, Biomarkers, Head and Neck Neoplasms, C-Reactive protein, Kinetics

## Abstract

Purpose: First-line immune checkpoint blockade has improved the prognosis of recurrent and/or metastatic head and neck squamous cell carcinoma (R/M HNSCC), but response rates remain low. In this study, we aimed to investigate the prognostic value of CRP and its early kinetics to predict response and survival in R/M HNSCC. Methods: A total of 87 patients who received first-line pembrolizumab for R/M HNSCC were analyzed. Three-fold cross-validation was used to estimate cut-off points of CRP at baseline and on-treatment (day 40 ± 10). Treatment response and survival were analyzed according to early CRP kinetics. The neutrophil-to-lymphocyte ratio (NLR) was used as a benchmark for the prognostic performance of CRP. Results: On-treatment CRP below 2 mg/dl, 4x the upper limit of normal (ULN), was associated with increased overall survival (OS), while on-treatment CRP below 3 mg/dl (6x ULN) was correlated with a higher disease control rate (DCR) and increased progression-free survival (PFS). CRP flare-responders and CRP responders showed a higher DCR and longer PFS than CRP non-responders. An NLR above 6 was a negative prognosticator for progression. In multivariable analysis, on-treatment CRP prevailed as the only significant prognosticator for OS (HR: 4.97, CI95%: 2.18–11.32, p < 0.001) and PFS (HR: 2.07, CI95%: 1.07–3.99, p = 0.030). Conclusion: On-treatment CRP was identified as a prognostic biomarker for objective response and survival in R/M HNSCC patients receiving first-line pembrolizumab and could be easily incorporated into clinical practice as a widely available and cost-effective biomarker.

**Supplementary Information:**

The online version contains supplementary material available at 10.1007/s10637-023-01388-x.

## Introduction

Amidst the advent of immune checkpoint inhibitors (ICI) and their transformative effect on the landscape of oncological care, the palliative treatment of recurrent and/or metastatic head and neck squamous cell carcinoma (R/M HNSCC) has undergone considerable changes in recent years. Among 500.000 deaths per year attributable to head and neck cancer [1], patients presenting with R/M HNSCC face a particularly dim prognosis with overall survival (OS) rates of less than a year [2].

In 2019, pembrolizumab was granted FDA approval as a single-agent, first-line treatment of R/M HNSCC in patients with a combined positive score (CPS) ≥ 1, enabling physicians for the first time to offer treatment-naïve, platinum-sensitive R/M HNSCC patients a non-chemotherapy-based regimen. In patients not amenable to monotherapy, pembrolizumab may be administered with concurrent chemotherapy (cisplatin or carboplatin plus 5-fluorouracil) [3].

Although the CPS, based on programmed death 1 ligand 1 (PD-L1) expression on both tumor and immune cells, has offered some utility in identifying patients with a higher likelihood of response to pembrolizumab [3], it is currently still deemed insufficient in predicting clinical benefit. A long-term follow-up analysis of KEYNOTE-012 has called into question the validity of excluding patients based on PD-L1 scoring since a clinically relevant 9% of patients with PD-L1 negative tumors still responded to pembrolizumab [4]. From a methodical standpoint, concerns related to low concordance between PD-L1 antibodies used for immunohistochemical staining [5] and PD-L1 status misclassification due to intratumoral heterogeneity have been raised [6].

Bloodborne biomarkers are easier to obtain and do not share the limitations of biopsy-driven methods arising from intratumoral heterogeneity and lack of easily repeatable sampling. In solid cancers, increasing evidence points towards the utility of widely available blood markers, such as the C-reactive protein (CRP), for prognosis [7]. Two studies recently demonstrated the prognostic power of early CRP kinetics, such as flare response, in metastatic renal cell carcinoma (mRCC) [8] and advanced non-small cell lung cancer (NSCLC) [9] as early as 4 weeks after ICI initiation. However, it is still unclear if CRP kinetics are superior in predicting outcomes compared to dichotomized CRP cut-off points.

To the best of our knowledge, we are the first to investigate CRP, and its kinetics, as prognosticators for survival and objective response in R/M HNSCC patients receiving first-line pembrolizumab. We sought to compare the performance of the CRP kinetics classification, as proposed by Fukuda et al. [8], to dichotomized cut-off points. As previous studies have implicated the early on-treatment neutrophil-to-lymphocyte ratio (NLR) as a biomarker for response and survival in ICI-treated R/M HNSCC [10, 11], we used the NLR as a benchmark for CRP’s prognostic performance.

## Materials and methods

### Study population

One hundred thirty-three patients, who received pembrolizumab for R/M HNSCC as palliative treatment between 2016 and 2021 at the Vienna General Hospital (Medical University of Vienna), were identified. Forty-seven patients did not meet the inclusion criteria. Therefore, 87 patients were included in the final analysis. A detailed flowchart for patient selection can be found in Supplementary Fig. 1. This study was approved by and conducted according to the ethical standards of the ethics committee of the Medical University of Vienna (approval number: 1324/2022).

### Study design

Patients received pembrolizumab once every 3 weeks (200 mg) or every 6 weeks (400 mg). A subset of patients received pembrolizumab and concurrent chemotherapy with cisplatin or carboplatin plus 5-fluorouracil (5-FU). Laboratory testing, including blood count, clinical chemistry, and immunological markers, was performed before the start of treatment and before every subsequent cycle. At baseline, only labs within 14 days of treatment initiation were eligible. On-treatment labs were collected on day 40 ± 10 to assure all patients received the same cumulative dose of pembrolizumab. For CRP kinetics analysis, patients were assigned to three groups based on the criteria of two recent studies describing CRP kinetics in metastatic renal cell carcinoma and non-small cell lung cancer [8, 9]. CRP flare-responders were defined as patients with a ≥ 100% increase in CRP by day 20 ± 10 and a return to baseline or lower by day 40 ± 10. CRP responders were defined as patients with a ≥ 30% decrease in CRP from baseline by day 40 ± 10 without prior flare. CRP non-responders comprised all patients not classified as CRP flare-responders or CRP responders.

Objective response according to the Response Evaluation Criteria in Solid Tumours (RECIST) version 1.1 was evaluated by CT and/or MRI studies of the head, neck, chest, and abdomen as clinically indicated [12]. Best overall response (bOR) was defined as the best objective response achieved during treatment. Patients with clear signs of progression at clinical examination or patients who died before the first restaging study were considered as progressive disease (PD).

The primary outcome of this study was OS. The secondary outcome of this study was PFS and the disease-control rate (DCR). OS was calculated from the start of treatment until death of any cause. PFS was defined as the start of treatment until progression according to imaging studies, clinical examination, or death of any cause if no progression was determined. The DCR was calculated as the percentage of patients with complete response (CR), partial response (PR), or stable disease (SD) as their bOR.

### Statistical analysis

Baseline and on-treatment CRP (mg/dl) and NLR levels, as well as absolute change between the two time points, were tested for Gaussian distribution using the Shapiro-Wilk test. Since our data uniformly followed non-Gaussian distributions, non-parametric tests were used for further analysis. Baseline and on-treatment levels were compared using the Wilcoxon test for paired samples. The absolute change was analyzed using the Mann-Whitney test for unpaired samples. CRP and NLR levels according to bOR were compared using the Kruskal-Wallis test with multiple comparisons using Dunn’s correction between PD and SD, PR and CR, respectively.

In lack of a validation cohort, we sought to cross-validate our baseline and on-treatment CRP and NLR cut-off points to improve their performance in external data sets. To this end, we used the cvauroc STATA module [13] (https://github.com/migariane/cvAUROC) and performed a 3-fold cross-validation with 10 repeats per fold for all cut-off points. Optimal cut-off points for overall survival and progression were selected by maximizing the Youden-Index [14]. Cut-off points with a cross-validated (cv) area under the operating curve (AUROC) of less than 0.75 were deemed not clinically relevant and were excluded from the analysis [10]. cvAUROC, cvSensitvity, and cvSpecificity were defined as the median AUROC, sensitivity, and specificity across all folds and repeats. Individual values are graphed in Supplementary Figs. 2 & 3 for CRP and NLR, respectively. Furthermore, 95% confidence intervals (CI95%) of the median for cvAUROC, cvSensitivity, and cvSpecificity were calculated.

Chi-squared or Fisher-exact testing was used for patient characteristics and bOR for baseline and on-treatment CRP and NLR, as well as CRP kinetics. Fisher-exact testing was used in cases where one or more cell counts were below 5.

Survival analysis was performed using the Kaplan-Maier survivor function and log-rank testing for OS and PFS. Additionally, univariable and multivariable Cox proportional hazard models were used to determine hazard ratios for OS and PFS. The multivariable model included all clinically relevant cut-off points for CRP and NLR, CRP kinetics, potential confounders that yielded significant results in univariable analysis, and presence or absence of concurrent chemotherapy.

## Results

### Patient characteristics

Out of 87 patients who received pembrolizumab as first-line treatment for R/M HNSCC, 56 patients were treated with monotherapy, and 31 patients underwent concurrent chemotherapy with cisplatin or carboplatin and 5-FU. Our study population had a mean age of 65 (range: 28–93) and was predominantly male (74%). The most common location of the primary was the oral cavity (n = 39), followed by the oropharynx (n = 18), of which 7 were HPV-associated. The cohort was evenly split between patients who presented with recurrent locoregional disease alone (49%) and patients with radiologically verified distant metastasis (51%), either with or without evidence of locoregional disease. Patients with distant metastasis comprised 42 cases with organ metastasis and 14 cases with distant lymph node metastasis.

Among those receiving monotherapy, 44 patients received 200 mg every 3 weeks, while 12 received 400 mg every 6 weeks. All patients undergoing concurrent chemotherapy received 200 mg every 3 weeks. The CPS score was available in 80 patients and was uniformly ≥ 1. The Eastern Cooperative Oncology Group performance status (ECOG PS) was assessed in all patients, with 49% of patients receiving a score of 0 and 51% scoring ≥ 1. Prior to pembrolizumab, 43% of patients had received systemic chemotherapy or targeted therapy agents (cisplatin/carboplatin or cetuximab) concurrently with radiotherapy. After pembrolizumab, 46% of patients had received at least one other chemotherapy regimen.

**Table 1:** Patient characteristics according to pembrolizumab regimen. Pembrolizumab monotherapy and pembrolizumab with concurrent chemotherapy were compared using Chi-squared or Fisher exact test

Characteristics	Total n (%)	Pembrolizumab monotherapy n (%)	Pembrolizumab + Platin + 5-FU n (%)	p-value
**Number of patients**	87	56	31	-
**Age in years, mean (range)**	65 (28–93)	68 (46–93)	59 (28–72)	-
≤ 65	49 (56%)	23 (41%)	26 (84%)	Chi-squared
> 65	38 (44%)	33 (59%)	5 (16%)	**< 0.001**
**Sex**				
Male	64 (74%)	39 (70%)	25 (81%)	Chi-squared
Female	23 (26%)	17 (30%)	6 (19%)	0.265
**Primary site**				
Oral cavity	39 (45%)	26 (46%)	13 (42%)	
Oropharynx	18 (21%)	11 (20%)	7 (22%)	
Hypopharynx	12 (14%)	6 (11%)	6 (19%)	
Larynx	10 (11%)	6 (11%)	4 (13%)	Fisher exact
Others^a^	8 (9%)	7 (12%)	1 (4%)	0.535
**HPV Status (oropharynx, n = 18)**				
p16 positive	7 (39%)	6 (55%)	1 (14%)	Fisher exact
p16 negative	11 (61%)	5 (45%)	6 (86%)	0.151
**Disease extent**				
Locoregional	43 (49%)	28 (50%)	15 (48%)	
Distant metastasis	10 (12%)	8 (14%)	2 (6%)	Fisher exact
Locoregional + distant metastasis	34 (39%)	20 (36%	14 (46%)	0.485
**Metastatic sites (organs)**				
0	45 (52%)	29 (52%)	16 (52%)	Chi-squared
≥ 1	42 (48%)	27 (48%)	15 (48%)	0.988
**Metastatic sites (distant lymph nodes)**				
0	73 (84%)	45 (80%)	28 (90%)	Fisher exact
≥ 1	14 (16%)	11 (20%)	3 (10%)	0.361
**Pembrolizumab dosage**				
200 mg every 3 weeks	75 (86%)	44 (79%)	31 (100%)	Fisher exact
400 mg every 6 weeks	12 (14%)	12 (21%)	0 (0%)	**0.004**
**CPS score**				
1–19	36 (41%)	15	21	Chi-squared
≥ 20	44 (51%)	34	10	**0.001**
*unknown*	*7 (8%)*	*7*	*0*	***-***
**ECOG PS**				
0	43 (49%)	22 (39%)	21 (68%)	
1	28 (32%)	20 (36%)	8 (25%)	Fisher exact
≥ 2	16 (19%)	14 (25%)	2 (7%)	**0.024**
**Prior systemic agents**				
RT with concurrent platin	27 (31%)	13 (23%)	14 (45%)	
RT with concurrent cetuximab	10 (12%)	3 (9%)	5 (16%)	Fisher exact
No prior systemic agents	50 (57%)	38 (68%)	12 (39%)	**0.030**
**Subsequent chemotherapy regimens**				
0	47 (54%)	32 (57%)	14 (45%)	Chi-squared
≥ 1^b^	40 (46%)	24 (43%)	17 (55%)	0.284
**Best overall response**				
CR	6 (7%)	5 (9%)	1 (3%)	
PR	28 (32%)	11 (20%)	17 (55%)	
SD	13 (15%)	8 (14%)	5 (16%)	Chi-squared^c^
PD	40 (46%)	32 (57%)	8 (26%)	**0.005**

^a^ Pembrolizumab monotherapy: Sinonasal = 4 (5%), Salivary glands = 2 (2%), Multifocal = 1 (2%; hypopharynx + larynx) / Pembrolizumab + Platin + 5-FU: Multifocal = 1 (4%; oropharynx + hypopharynx)

^b^ The most frequent subsequent regimens were Paclitaxel/Cetuximab (16 patients) and Docetaxel/Cetuximab (12 patients)

^c^ CR, PR, SD vs. PD

Abbreviations: 5-FU, 5-fluorouracil; CPS, combined positive score; CR, complete response; ECOG PS, Eastern Cooperative Oncology Group performance status; HPV, human papillomavirus; PD, progressive disease; PR, partial response; RT, radiotherapy; SD, stable disease

The bOR was assessed as defined by RECIST 1.1 criteria. In total, 46 patients exhibited PD, while 13, 28, and 6 patients showed CR, PR, and SD, respectively, resulting in a DCR of 53% and an ORR of 39%. Patients receiving pembrolizumab with concurrent chemotherapy were significantly younger, had a lower CPS and ECOG PS, received prior systemic agents more frequently, and had a higher DCR than patients who received single-agent pembrolizumab. (Table 1) The two regimens had no significant differences in baseline and on-treatment CRP or NLR levels. (Supplementary Table 1)

The median OS and PFS were 12.0 months and 3.9 months, respectively. There was no significant difference between patients receiving monotherapy and concurrent chemotherapy (median OS: 12.0 months vs. 13.0 months, p = 0.898; median PFS: 3.2 months vs. 5.6 months, p = 0.114).

### Cross-validated cut-off estimation and CRP kinetics

We aimed to determine clinically useful cut-off points for CRP and NLR. In lack of a validation cohort, we used 3-fold cross-validation with 10 repeats per fold to avoid overfitting of our model and to improve prognostic power in external cohorts (Supplementary Figs. 2 & 3). Cut-off points with a cvAUROC of less than 0.75 were considered not clinically relevant. For OS, on-treatment CRP at 2.0 mg/dl emerged as a clinically useful cut-off point (cvAUROC: 0.80), while the calculated on-treatment NLR cut-off point was deemed not sufficiently discriminatory (cvAUROC: 0.71). For disease progression, both on-treatment CRP at 3.0 mg/dl (cvAUROC: 0.77) and on-treatment NLR at 6.0 (cvAUROC: 0.76) showed clinical relevance. Baseline CRP and NLR did not meet the requirements for adequate prognostic power (cvAUROC < 0.75) and were therefore excluded from further analysis. (Supplementary Table 2)

Next, we aimed to determine the clinical utility of early CRP kinetics to predict survival and response. Patients were, therefore, split into three groups: CRP responders (CRP decrease of ≥ 30% by day 40 ± 10 days), CRP flare-responders (CRP increase of ≥ 100% by day 20 ± 10 followed by a return to or below baseline by day 40 ± 10) and CRP non-responders (all other patients). Overall, 19 patients were classified as responders, 8 as flare-responders, and 52 as non-responders. (Supplementary Fig. 4) Responders and flare-responders were significantly more common in patients with concurrent chemotherapy compared to monotherapy (57% vs. 20%, p = 0.003).

### Best overall response

**Fig. 1:**
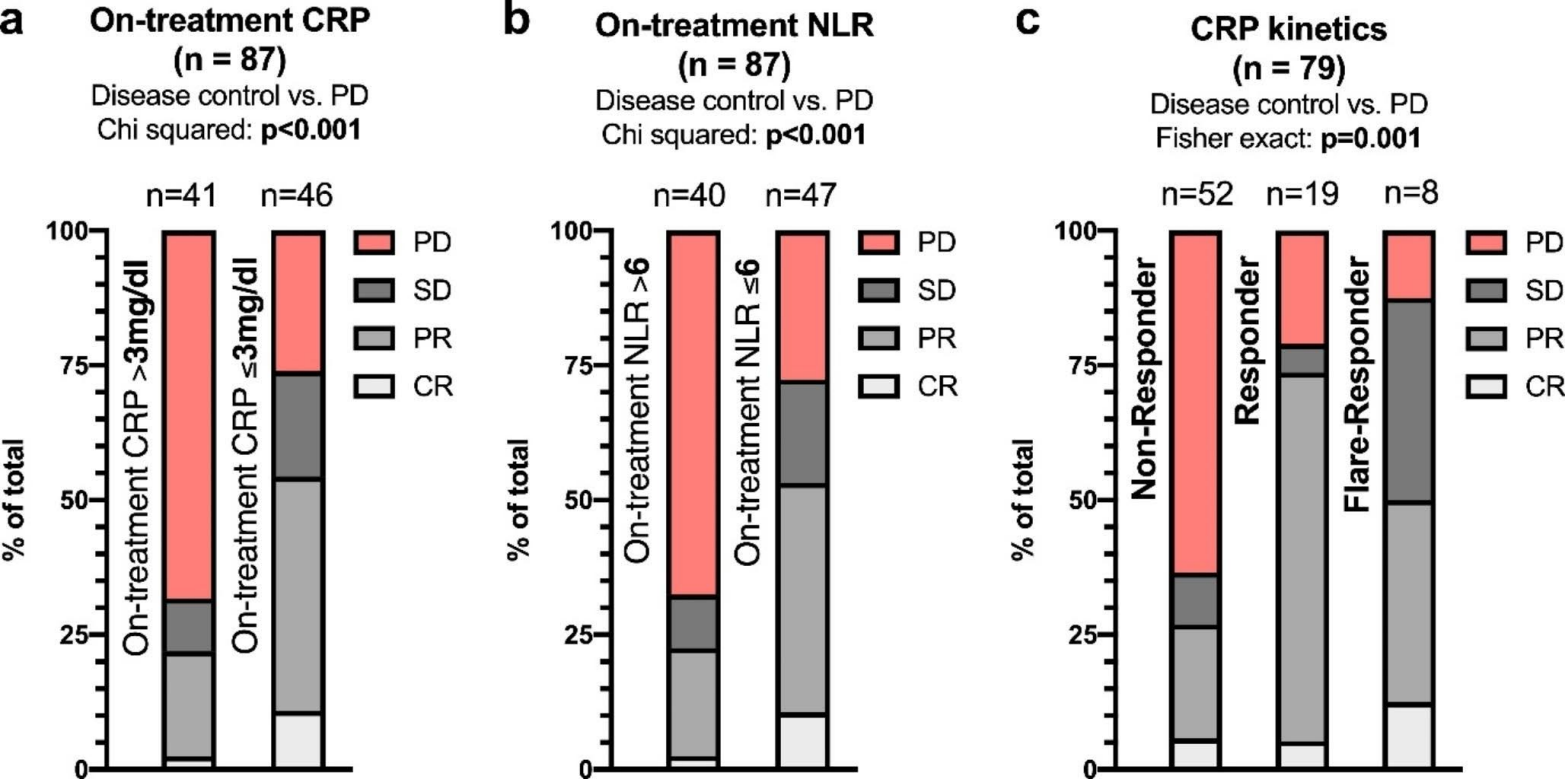
bOR according to on-treatment CRP **(a)**, on-treatment NLR **(b)** and CRP kinetics **(c)**. The difference in disease control (SD, PR or CR) between groups was compared using the Chi-squared or Fisher exact test. Abbreviations: bOR, best overall response; CR, complete response; CRP, C-reactive protein; NLR, neutrophil-to-lymphocyte ratio; PD, progressive disease; PR, partial response; SD, stable disease

After defining clinically relevant cut-off points for progression, we analyzed their performance according to bOR. (Fig. 1) Patients with on-treatment CRP levels above 3 mg/dl presented with higher PD rates than to those with CRP levels below 3 mg/dl (68% vs. 26%, p < 0.001). Similarly, on-treatment NLR levels above 6 were associated with higher rates of PD (66% vs. 28%, p < 0.001). For CRP kinetics, CRP non-responders showed higher rates of PD compared to CRP responders and CPR flare-responders, respectively (63% vs. 21% vs. 13%, p = 0.001). During subgroup analysis, the higher PD ratio remained significant for all three markers in patients receiving pembrolizumab monotherapy, while there was no significant difference for patients with concurrent chemotherapy. (Supplementary Fig. 5)

**Fig. 2:**
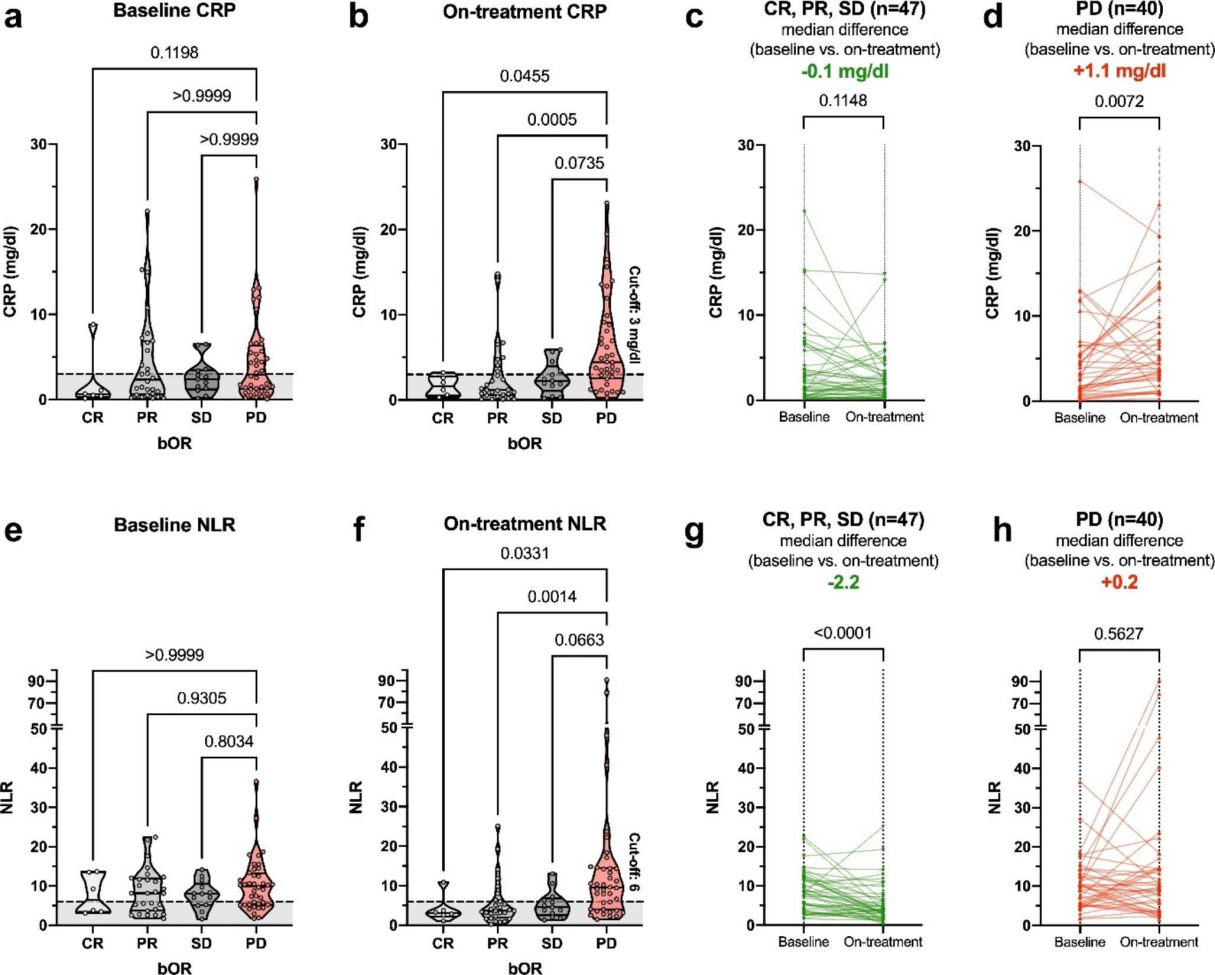
CRP and NLR levels according to bOR and time points. CRP (**a, b**) and NLR (**e, f**) levels are shown according to the bOR at baseline and on-treatment (day 40 ± 10). PD was compared to SD, PR and CR, respectively, employing Kruskal-Wallis testing and multiple comparison using Dunn’s correction. The cross-validated on-treatment cut-off points for progression (CRP: 3 mg/dl; NLR: 6) are shown as a dashed line on baseline and on-treatment graphs. Kinetics of baseline and on-treatment CRP (**c, d**) and NLR levels (**g, h**) split according to patients presenting with PD and disease control (CR, PR, SD) are shown as individual line plots. CRP and NLR level differences between the two time points were compared by the Wilcoxon test for paired samples. Abbreviations: bOR, best overall response; CR, complete response; CRP, C-reactive protein; NLR, neutrophil-to-lymphocyte ratio; PD, progressive disease; PR, partial response; SD, stable disease

Next, we sought to analyze differences in CRP and NLR levels according to bOR at the baseline and on-treatment time points. (Fig. 2) Baseline CRP and NLR levels were not significantly different between patients presenting with PD and CR, PR or SD, respectively. On-treatment, CRP levels were significantly higher for patients with PD compared to CR (median: 4.4 mg/dl vs. 1.6 mg/dl, p = 0.046) and PR (median: 4.4 mg/dl vs. 1.2 mg/dl, p < 0.001). Similarly, on-treatment NLR levels were significantly lower for CR (median: 3.1 vs. 9.6, p = 0.033) and PR (median: 3.7 vs. 9.6, p = 0.001) compared to PD. When comparing the baseline and on-treatment time point, CRP significantly increased in patients with PD (median: +1.1 mg/dl, p = 0.007), while NLR showed a significant decrease in patients with CR, PR or SD (median: -2.2, p < 0.001).

### Survival

**Fig. 3:**
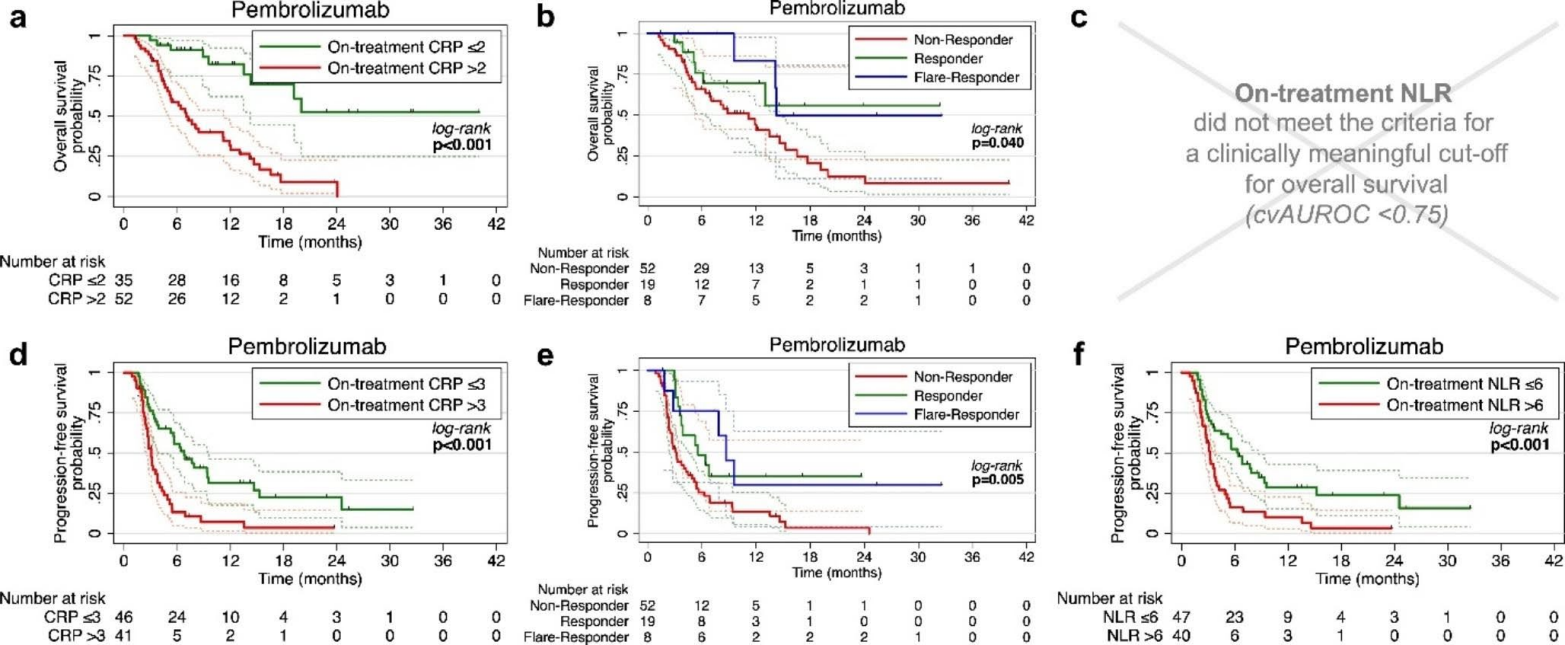
Survival analysis. Kaplan-Meier plots with CI95% (dashed lines) for OS and PFS dichotomized by the cross-validated cut-off points for on-treatment CRP in mg/dl (**a, d**), CRP kinetics (**b, e**), on-treatment NLR (**c, f**) levels are shown. Log-rank testing was used for comparison of the survival curves. Abbreviations: CI95%, 95% confidence interval; CRP, C-reactive protein; cvAUROC, cross-validated area under the receiver operating curve; NLR, neutrophil-to-lymphocyte ratio; OS, overall survival; PFS, progression-free survival

Finally, we investigated the prognostic potential of our markers for OS and PFS. (Fig. 3) One-year OS was higher in patients with on-treatment CRP below 2 mg/dl (82.5% vs. 31.8%, p < 0.001) and in CRP flare-responders and CPR responders compared to CRP non-responders (83.3% vs. 69.1% vs. 44.1%, p = 0.040). Univariable analysis revealed ECOG performance status and on-treatment CRP as significant prognosticators for OS. (Supplementary Table 3) In our multivariable model, on-treatment CRP remained as the only prognosticator for OS with a hazard ratio (HR) of 4.97 (CI95%: 2.18–11.32, p < 0.001). (Table 2)

Correspondingly, one-year PFS was longer in patients with on-treatment CRP below 3 mg/dl (31.4% vs. 7.1%, p < 0.001) and in CRP flare-responders and CRP responders compared to CRP non-responders (30.6% vs. 34.3% vs. 13.6%, p = 0.005). Furthermore, an on-treatment NLR below 6 was a favorable prognosticator (one-year OS: 28.7% vs. 10.1%, p < 0.001). Univariable analysis revealed on-treatment CRP and on-treatment NLR, as well as CRP responder and CRP flare-responder status as significant prognosticators. However, on-treatment CRP again emerged as the only prognostic factor for PFS in our multivariable model with an HR of 2.07 (1.07–3.99, p = 0.030).

During sub-group analysis, on-treatment CRP below 2 mg/dl prevailed as a prognosticator for OS both in patients receiving pembrolizumab monotherapy (one-year OS: 84.2% vs. 32.0%, p < 0.001) and pembrolizumab with concurrent chemotherapy (one-year OS: 77.9% vs. 32.4%, p = 0.011). (Supplementary Fig. 6) Pertaining to PFS, both on-treatment CRP below 3 mg/dl (one-year OS - monotherapy: 35.8% vs. 7.7%, p = 0.003 / concurrent chemotherapy: 20.0% vs. 0.0%, p = 0.007) and on-treatment NLR below 6 (one-year OS - monotherapy: 30.3% vs. 14.3%, p = 0.035 / concurrent chemotherapy: 21.2% vs. 0.0%, p = 0.004), remained as favorable prognosticators. (Supplementary Fig. 7) CRP kinetics yielded no significant prognostic value for OS or PFS when analyzed individually by regimen.

**Table 2:** Multivariable analysis – A reduced model for OS and PFS.

	Multivariable – OS Reduced model			Multivariable – PFS Reduced model		
Variables	n	HR (CI95%)	p	n	HR (CI95%)	p
**Concurrent chemotherapy** Present vs. absent (ref)	79	1.26 (0.64–2.47)	0.498	79	0.9 (0.51–1.59)	0.714
**ECOG PS** ≥ 1 vs. 0 (ref)	79	1.46 (0.78–2.76)	0.240	-	-	-
**On-treatment CRP**						
> 2 mg/dl vs. ≤2 mg/dl (ref)	79	4.97 (2.18–11.32)	**< 0.001**	-	-	-
> 3 mg/dl vs. ≤3 mg/dl (ref)	-	-	-	79	2.07 (1.07–3.99)	**0.030**
**On-treatment NLR** > 6 vs. ≤6 (ref)	-	-	-	79	1.18 (0.61–2.26)	0.620
**CRP kinetics**	79			79		
Non-responder (ref)		ref	ref		ref	ref
Responder		0.51 (0.21–1.28)	0.152		0.55 (0.28–1.08)	0.083
Flare-responder		0.59 (0.17–2.10)	0.415		0.46 (0.17–1.29)	0.140

Abbreviations: CI95%, 95% confidence interval; CPS, combined positive score; CRP, C-reactive protein; ECOG PS, Eastern Cooperative Oncology Group performance status; HR, hazard ratio; NLR, neutrophil-to-lymphocyte ratio; OS, overall survival; PFS, progression-free survival; ref, reference; RIT, radioimmunotherapy

## Discussion

In this study, we showed the prognostic significance of elevated on-treatment CRP levels and CRP kinetics for objective response and survival in R/M HNSCC patients receiving first-line pembrolizumab with or without concurrent chemotherapy. We, furthermore, validated previous findings regarding the on-treatment NLR as a biomarker for progression.

Previous studies have investigated the modified Glasgow prognostic score and the CRP-to-albumin ratio in R/M HNSCC patients receiving second-line nivolumab [15–17]. However, the utility of CRP as an individual biomarker in predicting response and survival, especially in the first-line setting of R/M HNSCC, has been unclear. Other blood-based biomarkers, such as the NLR, have previously been implicated as a prognosticator for OS and PFS in R/M HNSCC receiving ICI. At the baseline time point, the NLR has been found to predict survival outcomes in two previously reported patient cohorts: one treated with second-line nivolumab [17] and another treated with mixed ICI regimens [10]. However, using a cross-validated approach to cut-off estimation, we found both baseline CRP and baseline NLR to not be sufficiently discriminatory in a clinically meaningful way (cvAUROC < 0.75). Our study, therefore, does not support the use of CRP or NLR as a baseline biomarker for ICI response in first-line pembrolizumab.

However, at the on-treatment time point (day 40 ± 10), we demonstrated that CRP levels above 2 mg/dl, or 4x the upper limit of normal (ULN), and above 3 mg/dl, or 6x ULN, are negative prognosticators for overall survival and progression-free survival, respectively. Regarding objective response, patients with CRP levels above 3 mg/dl at the on-treatment time point presented with significantly higher rates of progressive disease. The NLR acted as a benchmark based on previous reports of its utility as a bloodborne biomarker for ICI response in R/M HNSCC [10, 11]. The on-treatment NLR (cvAUROC = 0.71) did not meet our criteria for a clinical meaningful biomarker for OS, while on-treatment CRP (cvAUROC = 0.80) did. In our multivariable analysis for PFS, which included the on-treatment NLR as a confounder, on-treatment CRP was the only marker to remain significant. Based on these findings, we conclude that on-treatment CRP is a more consistent and better-performing biomarker in R/M HNSCC patients receiving first-line pembrolizumab compared to the NLR.

In addition to on-treatment CRP, we are the first to describe the prognostic value of CRP flare-response in R/M HNSCC patients receiving ICIs. Fukuda et al. [8] initially proposed a CRP kinetics-based classification system consisting of CRP flare-responders, CRP responders, and CRP non-responders to predict objective response and PFS in patients receiving nivolumab for mRCC. Their classification considered a time frame of 3 months, by which point the first restaging study is likely to have already happened, thereby narrowing its clinical utility. The second report based on this classification in a cohort of NSCLC patients receiving ICIs described a prognostic significance of flare response as early as 4 weeks [9].

In clinical reality, the shortest feasible time frame to determine CRP kinetics, including flare-response for R/M HNSCC, is at 6 weeks since blood draws are usually performed at baseline and then once every 3 weeks shortly before ICIs are administrated. We, therefore, adapted our time frame for CRP kinetics to 6 weeks. In our study, CRP flare-responders and CRP responders achieved longer PFS than CRP non-responders in univariable analysis, and their disease control rate was significantly higher. Our univariable analysis did not show any correlation between OS and CRP kinetics. CRP kinetics did not remain a significant prognosticator for PFS in our multivariable model. Consequently, our data indicate that CRP kinetics offer an exciting glimpse into the immunological underpinnings of ICI response. However, on-treatment CRP is likely a more consistent prognostic biomarker for survival and objective response.

On-treatment CRP further remained a significant prognosticator in subgroup analysis for OS and PFS of patients receiving pembrolizumab monotherapy and pembrolizumab with concurrent chemotherapy. As a biomarker for bOR, our findings support elevated on-treatment CRP above 3 mg/dl as an indicator for progressive disease in the pembrolizumab monotherapy cohort but not in the concurrent chemotherapy cohort. Despite a similar trend to the monotherapy cohort, the lack of significance may have resulted from the smaller number of patients and the higher disease control rate in the concurrent chemotherapy cohort.

One might argue that baseline biomarkers are preferable to on-treatment markers for their potential application in aiding treatment choice. However, in the realm of ICI, on-treatment markers also yield substantial utility. Our on-treatment time point around 6 weeks into treatment opens a considerable therapeutic window until the first restaging 8–12 weeks after treatment initiation and another 4–8 weeks until a potential second imaging study in suspected pseudo-progression cases according to iRECIST [18] As such, low on-treatment CRP could be factored into consideration for treatment de-escalation, such as early discontinuation of concurrent chemotherapy. Conversely, high on-treatment CRP could potentially inform an early transition to second-line treatment regimens for patients that do not respond to pembrolizumab.

In further support of on-treatment markers, recent studies have indicated that patients who appear to not clinically benefit from ICIs respond better to subsequent salvage chemotherapy regimens in several cancer types, including head and neck cancer [19, 20]. Baseline markers may preclude R/M HNSCC patients from receiving first-line ICIs that could potentially sensitize them to a subsequent chemotherapy-based regimen. With on-treatment markers, such as CRP, being factored into the clinical decision-making process, patients could still receive up to two doses of pembrolizumab before being taken off treatment to initiate salvage chemotherapy that could benefit from ICI induction. However, further studies will be necessary to elucidate the role of ICIs as potential chemosensitizers in the palliative setting of R/M HNSCC.

From a pathophysiological standpoint, the underlying causality for the prognostic performance of on-treatment CRP elevation is likely two-fold. For one, CRP has been described as an indicator of tumor burden [21] and, as such, may act as a surrogate marker for growing and progressing tumors eliciting a stronger immunological response accompanied by CRP elevation. On the other hand, recent findings showed that elevated levels of CRP impaired adaptive immunity and propagated immunosuppression in patients with metastatic melanoma [22]. Our observation that patients with PD had a median CRP increase of 1.1 mg/dl by day 40 ± 10, while median CRP levels remained steady in those with disease control, lends support to this narrative. Nevertheless, more studies are needed to draw solid conclusions on the causative link between CRP fluctuations and ICI response.

Our study has several limitations. The retrospective study design carries inherent limitations related to data availability and conformity. In the absence of a validation cohort due to the relative scarcity of R/M HNSCC compared to other more common malignancies, such as NSCLC, coupled with the recency of first-line pembrolizumab approval, we employed internal cross-validation to improve the performance and accuracy of our CRP and NLR cut-off points in external cohorts. Still, our results need to be validated in external data sets to further support the use of on-treatment CRP as a prognostic biomarker.

## Conclusion

This study is the first to describe the correlation between on-treatment CRP, objective response, and survival in R/M HNSCC patients treated with first-line pembrolizumab. Although of less prognostic value, CRP kinetics, including CRP flare response, was linked to progression, providing insights into the underlying immunological mechanisms of immune checkpoint blockade in R/M HNSCC. Prospective studies with a larger study population are needed to support the use of on-treatment CRP as a widely available, easy-to-obtain, and cost-effective prognosticator.

### Electronic supplementary material

Below is the link to the electronic supplementary material.

Supplementary Material 1: Tables & Figures

## Data Availability

The datasets generated and analyzed during the current study are available from the corresponding author on reasonable request.

## Abbreviations

ICI: immune checkpoint inhibitor
R/M HNSCC: recurrent and/or metastatic head and neck squamous cell carcinoma
OS: overall survival
PD-1: programmed death 1 receptor
CPS: combined positive score
PD-L1: programmed death 1 ligand 1
CRP: C-reactive protein
mRCC: metastatic renal cell carcinoma
NSCLC: non-small cell lung cancer
ORR: overall response rate
PFS: progression-free survival
NLR: neutrophil-to-lymphocyte ratio
5-FU: 5-fluorouracil
RECIST: Response Evaluation Criteria in Solid Tumours
bOR: best overall response
PD: progressive disease
DCR: disease control rate
CR: complete response
PR: partial response
SD: stable disease
Cv: cross-validated
AUROC: area under the receiver operating curve
CI95%: 95% confidence interval
ECOG PS: Eastern Cooperative Oncology Group performance status
HR: hazard ratio
ULN: upper limit of normal
