# Supplementary material for: Early on-treatment C-reactive protein and its kinetics predict survival and response in recurrent and/or metastatic head and neck cancer patients receiving first-line pembrolizumab
Source: Invest New Drugs. 2023 Aug 21;41(5):727–36. doi: 10.1007/s10637-023-01388-x (PMC10560194; doi:10.1007/s10637-023-01388-x)
Supplement: Supplementary file 1 — Supplementary Material 1: Tables & Figures [file 10637_2023_1388_MOESM1_ESM.docx]

**SUPPLEMENTARY MATERIAL**

*Investigational New Drugs*

**Early on-treatment C-reactive protein and its kinetics predict survival and response in recurrent and/or metastatic head and neck cancer patients receiving first-line pembrolizumab**

Markus Haas, MD BA^1^, Alexander Lein, BEng^1^, Thorsten Fuereder, MD^2^, Julia Schnoell, MD^1^, Faris F Brkic, MD PHD^1^, David T Liu, MD PHD^1^, Lorenz Kadletz-Wanke, MD PHD^1^, Gregor Heiduschka, MD^1^, Bernhard J Jank, MD^1^

^1^ Department of Otorhinolaryngology, Head and Neck Surgery, Medical University of Vienna, Vienna, Austria

^2^ Division of Oncology, Department of Medicine I, Medical University of Vienna, Vienna, Austria

**Corresponding author:**

Gregor Heiduschka

E-mail: gregor.heiduschka@meduniwien.ac.at

**Tables**

|  | **Total** | **Pembrolizumab**  **Monotherapy** | **Pembrolizumab**  **+ Platin + 5-FU** | **p-value** |
| --- | --- | --- | --- | --- |
|  | n = 87 | n = 56 | n = 31 | Mann-Whitney |
| **Baseline CRP, median (IQR)** | 2.38 (0.79-5,75) | 1.84 (0.67-5.41) | 2.53 (1.16-6.61) | 0.431 |
| **On-treatment CRP, median (IQR)** | 2.86 (0.97-5.67) | 3.19 (0.99-6.83) | 2.5 (0.90-4.10) | 0.195 |
| **Baseline NLR, median (IQR)** | 8.37 (5.14-12.29) | 7.92 (5.10-11.86) | 8.75 (5.29-12.49) | 0.455 |
| **On-treatment NLR, median (IQR)** | 5.32 (3.14-10.86) | 7.26 (3.19-12.45) | 4.11 (2.04-7.20) | 0.057 |
|  | n = 79 | n = 49 | n = 30 |  |
| **CRP kinetics** |  |  |  |  |
| Non-responder | 52 (66%) | 39 (80%) | 13 (43%) |  |
| Responder | 19 (24%) | 7 (14%) | 12 (40%) | Fisher exact |
| Flare-Responder | 8 (10%) | 3 (6%) | 5 (17%) | **0.003** |

**Supplementary Table 1.** Differences in CRP and NLR levels and CRP kinetics according to pembrolizumab regiment.

Abbreviations: CRP, C-reactive protein; IQR, interquartile rage; 5-FU, 5-fluorouracil; NLR, neutrophil-to-lymphocyte ratio.

|  | Median  (n=87) | **Cross-validated**  **cut-off point**  median (CI95%) | **Cross-validated**  **sensitivity**  median (CI95%) | **Cross-validated**  **specificity**  median (CI95%) | **Cross-validated**  **AUROC**  median (CI95%) |
| --- | --- | --- | --- | --- | --- |
| **Overall survival** |  |  |  |  |  |
| Baseline CRP (mg/dl) | 2.38 | 1.4 (1.3-2.5) | 65 (56-83) | 62 (41-72) | 0.67 (0.65-0.69) |
| On-treatment CRP (mg/dl) | 2.86 | **2.0 (1.8-2.9)** | **76 (73-83)** | **74 (69-77)** | **0.80 (0.79-0.81)** |
| Baseline NLR | 8.37 | 9.2 (5.6-10.1) | 67 (56-73) | 53 (44-62) | 0.52 (0.49-0.55) |
| On-treatment NLR | 5.32 | 5.5 (4.1-6.4) | 63 (58-67) | 74 (72-77) | 0.71 (0.69-0.73) |
| **Progression** |  |  |  |  |  |
| Baseline CRP (mg/dl) | 2.38 | 3.5 (2.1-4.0) | 55 (50-65) | 57 (49-74) | 0.59 (0.55-0.62) |
| On-treatment CRP (mg/dl) | 2.86 | **3.0 (2.6-3.4)** | **71 (68-73)** | **72 (70-74)** | **0.77 (0.75-0.79)** |
| Baseline NLR | 8.37 | 8.5 (5.6-10.1) | 60 (48-85) | 60 (32-72) | 0.60 (0.56-0.62) |
| On-treatment NLR | 5.32 | **6.0 (5.3-6.4)** | **68 (65-70)** | **78 (74-79)** | **0.76 (0.74-0.78)** |

**Supplementary Table 2.** Cross-validated cut-off estimation for CRP and NLR at baseline and on-treatment (day 40±10). Cut-off points deemed clinically relevant (cvAUROC >0.75) are highlighted in bold.

Abbreviations: AUROC, area under the receiver operating curve; CI95%, 95% confidence interval; CRP, C-reactive protein; cv, cross-validated; NLR, neutrophil-to-lymphocyte ratio.

|  | Univariable – OS | | | Univariable – PFS | | |
| --- | --- | --- | --- | --- | --- | --- |
| Variables | n | HR (CI95%) | p | n | HR (CI95%) | p |
| **Age (years)**  ≥65 vs. <65 (ref) | 87 | 1.01 (0.57-1.80) | 0.967 | 87 | 1.14 (0.70-1.83) | 0.603 |
| **Sex**  male vs. female (ref) | 87 | 1.11 (0.58-2.10) | 0.751 | 87 | 1.27 (0.75-2.14) | 0.370 |
| **HPV+OPSCC**  HPV+OPSCC vs. other primaries (ref) | 87 | 0.68 (0.24-1.89) | 0.456 | 87 | 1.08 (0.46-2.51) | 0.860 |
| **Disease extent**  distant metastasis vs. locoregional (ref) | 87 | 0.69 (0.39-1.24) | 0.219 | 87 | 0.71 (0.44-1.15) | 0.163 |
| **Distant lymph node metastasis**  Present vs. absent (ref) | 87 | 0.46 (0.18-1.15) | 0.097 | 87 | 0.50 (0.25-1.03) | 0.060 |
| **Distant organ metastasis**  Present vs. absent (ref) | 87 | 0.79 (0.44-1.40) | 0.414 | 87 | 0.76 (0.47-1.23) | 0.263 |
| **Concurrent chemotherapy**  Present vs. absent (ref) | 87 | 0.96 (0.52-1.76) | 0.898 | 87 | 0.67 (0.40-1.11) | 0.118 |
| **ECOG PS**  ≥1 vs 0 (ref) | 87 | 2.10 (1.17-3.75) | **0.013** | 87 | 1.57 (0.97-2.52) | 0.064 |
| **CPS score**  ≥20 vs 1-19 (ref) | 80 | 0.91 (0.50-1.66) | 0.755 | 80 | 1.06 (0.64-1.74) | 0.830 |
| **Prior CRT (platin)**  Received vs not-received (ref) | 87 | 1.45 (0.80-2.64) | 0.221 | 87 | 1.22 (0.74-2.03) | 0.431 |
| **Prior RIT (cetuximab)**  Received vs. not-received (ref) | 87 | 1.40 (0.62-3.14) | 0.418 | 87 | 1.48 (0.72-3.01) | 0.283 |
| **On-treatment CRP** |  |  |  |  |  |  |
| >2 mg/dl vs. ≤2 mg/dl (ref) | 87 | 5.18 (2.47-10.85) | **<0.001** |  | - | **-** |
| >3 mg/dl vs. ≤3 mg/dl (ref) | - | - | - | 87 | 2.74 (1.67-4.5) | **<0.001** |
| **On-treatment NLR**  >6 vs. ≤6 (ref) | - | - | - | 87 | 2.32 (1.43-3.77) | **0.001** |
| **CRP kinetics** | 79 |  |  | 79 |  |  |
| Non-responder (ref) |  | ref | ref |  | ref | ref |
| Responder |  | 0.46 (0.19-1.09) | 0.077 |  | 0.44 (0.23-0.86) | **0.016** |
| Flare-responder |  | 0.32 (0.10-1.06) | 0.062 |  | 0.33 (0.13-0.84) | **0.020** |

**Supplementary Table 3.** Univariable analysis for OS and PFS.

Abbreviations: CI95%, 95% confidence interval; HR, hazard ratio; ref, reference; CRP, C-reactive protein; CPS, combined positive score; CRT, chemoradiotherapy; ECOG PS, Eastern Cooperative Oncology Group performance status; HPV, human papillomavirus; NLR, neutrophil-to-lymphocyte ratio; OPSCC, oropharyngeal squamous cell carcinoma; OS, overall survival; PFS, progression-free survival; RIT, radioimmunotherapy.

**Figures**


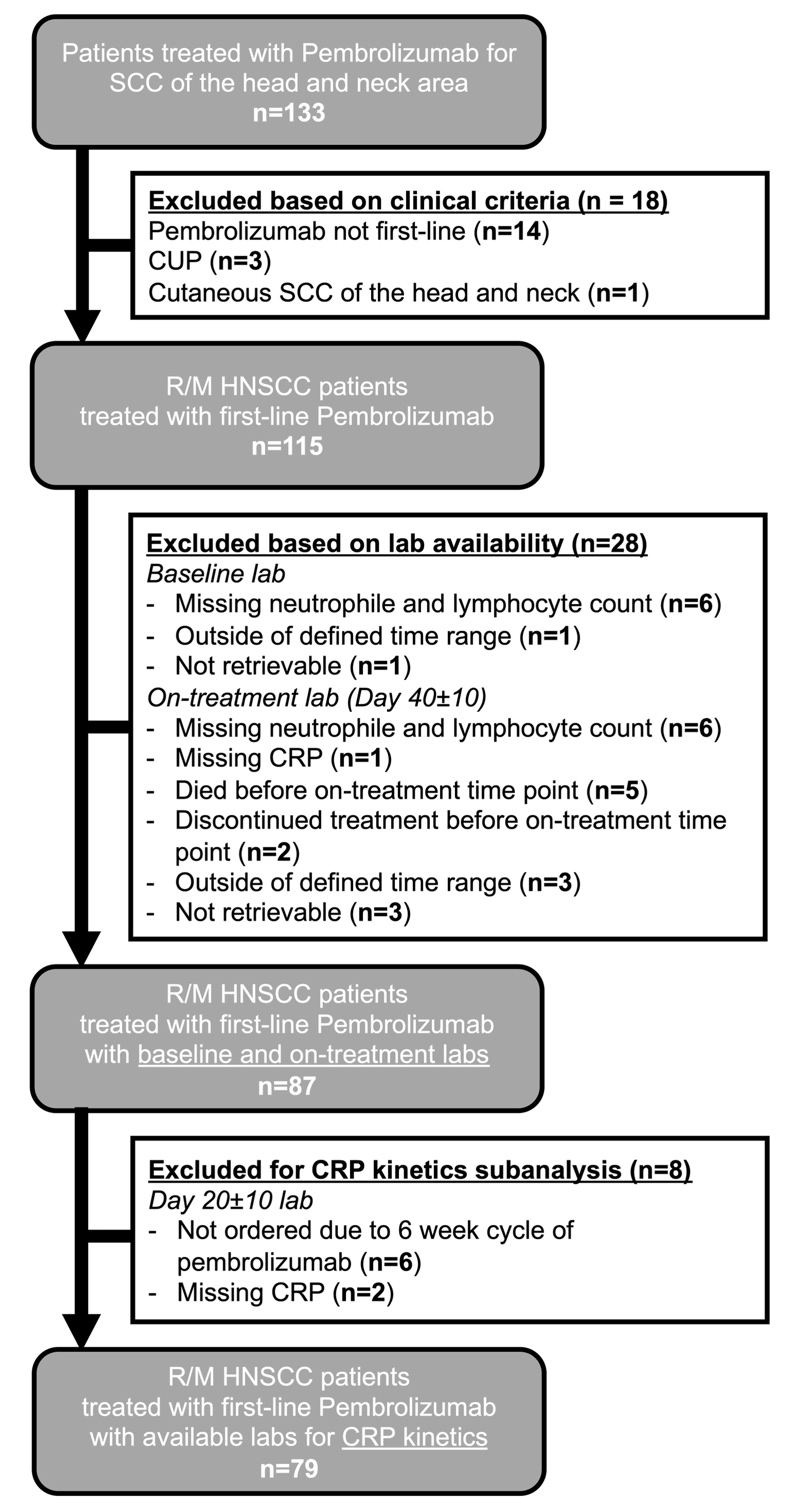


**Supplementary Fig. 1** Flow chart for patient selection.

Abbreviations: CRP, C-reactive protein; CUP, cancer of unknown primary; R/M HNSCC, recurrent and/or metastatic head and neck squamous cell carcinoma; SCC, squamous cell carcinoma.


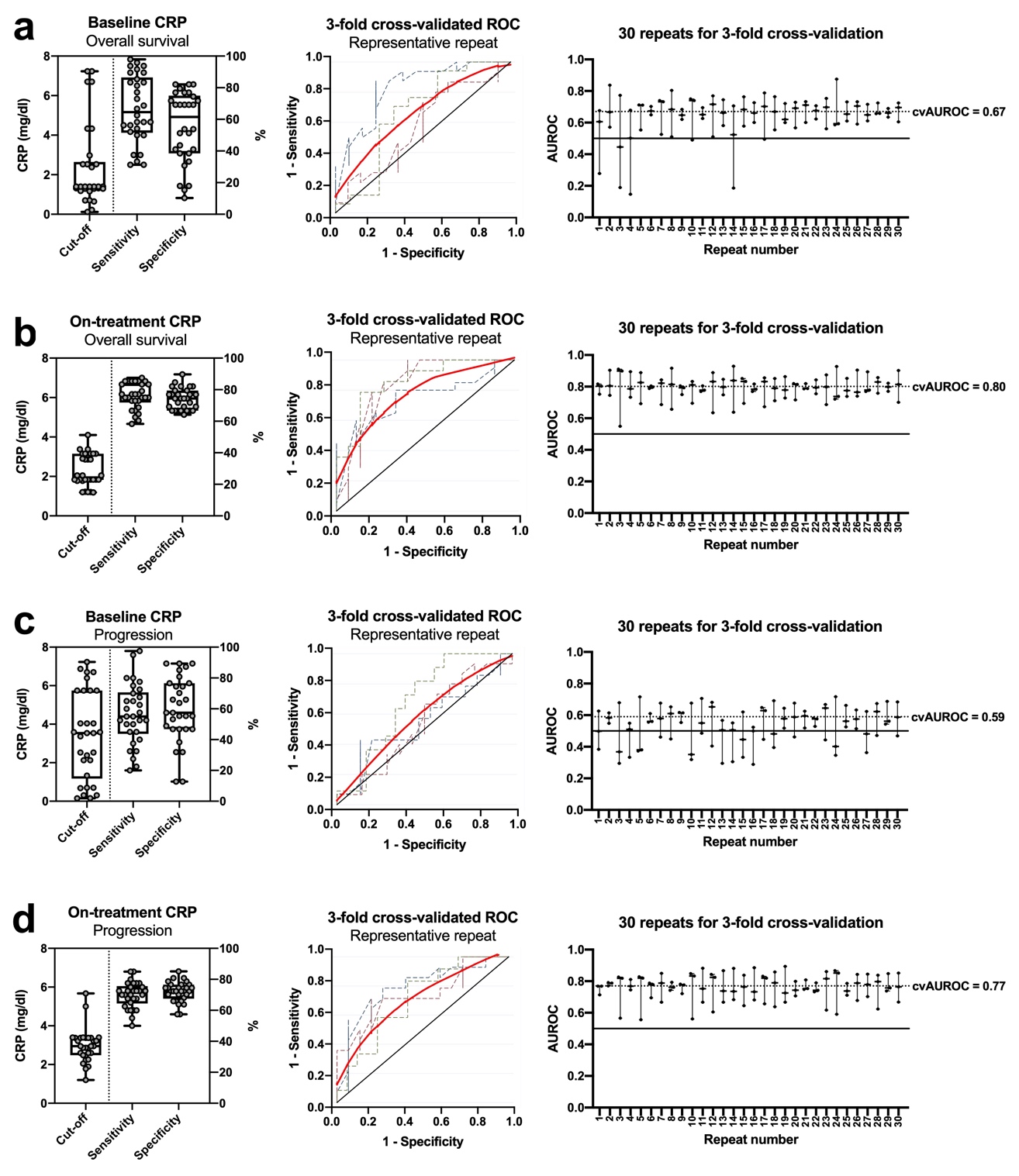


**Supplementary Fig. 2** Cross-validated cut-off estimation for baseline and on-treatment CRP for overall survival (a, b) and progression (c, d). Box-plots of individual values of estimated cut-offs for all 30 repeats of 3-fold cross-validation are shown in the first column. In the second column, a 3-fold cross-validated receiver operating curve of a representative repeat is shown. The area under the ROC of all three folds for every repeat (1-30) is shown in the third column with the cross-validated AUROC (median of all folds and repeats) illustrated as a dotted line.

Abbreviations: AUROC, area under the receiver operating curve; CRP, C-reactive protein; cv, cross-validated; NLR, neutrophil-to-lymphocyte ratio.


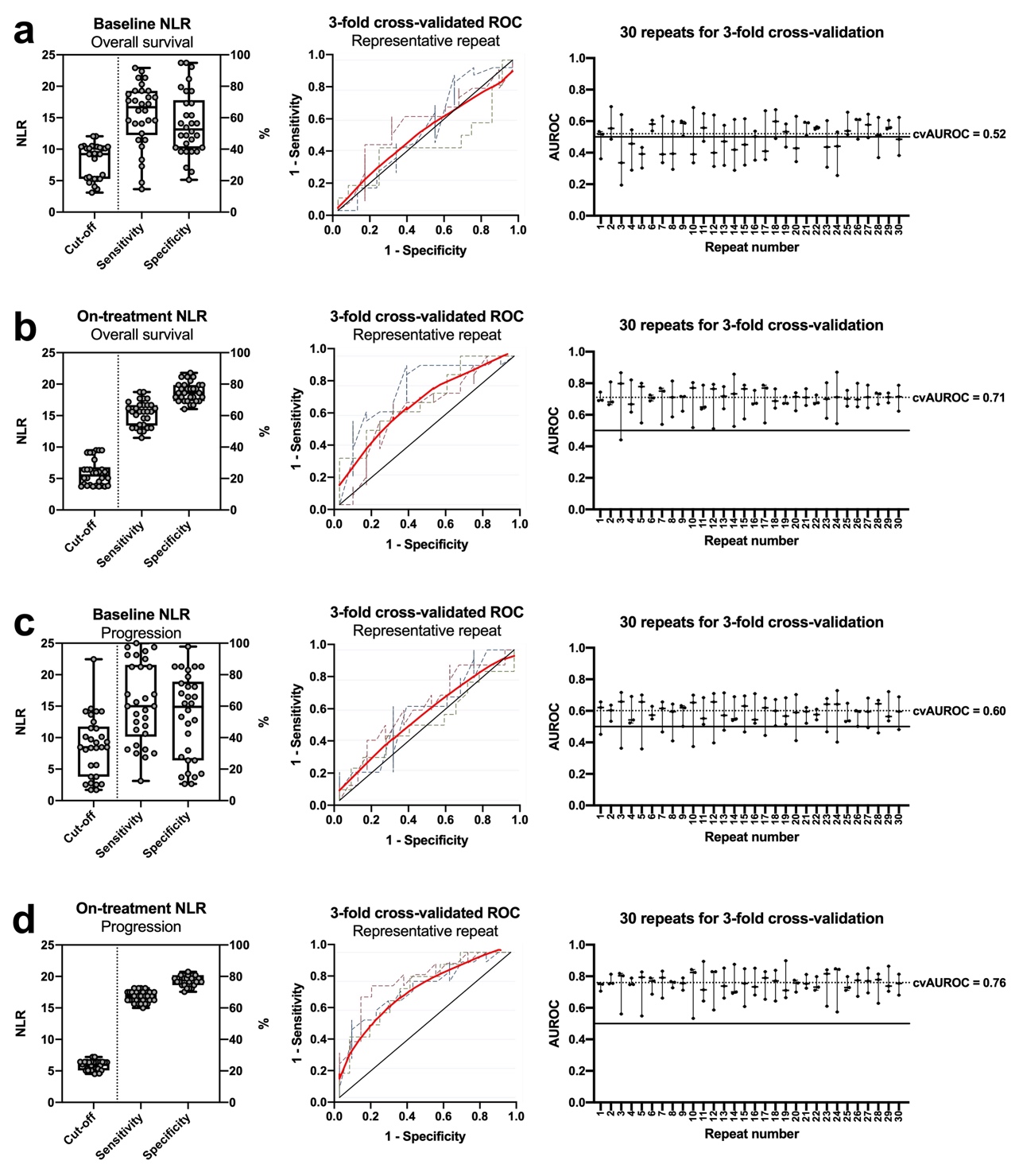


**Supplementary Fig. 3** Cross-validated cut-off estimation for baseline and on-treatment NLR for overall survival (a, b) and progression (c, d). Box-plots of individual values of estimated cut-offs for all 30 repeats of 3-fold cross-validation are shown in the first column. In the second column, a 3-fold cross-validated receiver operating curve of a representative repeat is shown. The dashed lines in the background represent ROCs of each fold, while the red continuous line is the smoothed average of all three folds. The area under the ROC of all three folds for every repeat (1-30) is shown in the third column with the cross-validated AUROC (median of all folds and repeats) illustrated as a dotted line.

Abbreviations: AUROC, area under the receiver operating curve; CRP, C-reactive protein; cv, cross-validated; NLR, neutrophil-to-lymphocyte ratio; ROC, receiver operating curve.

**
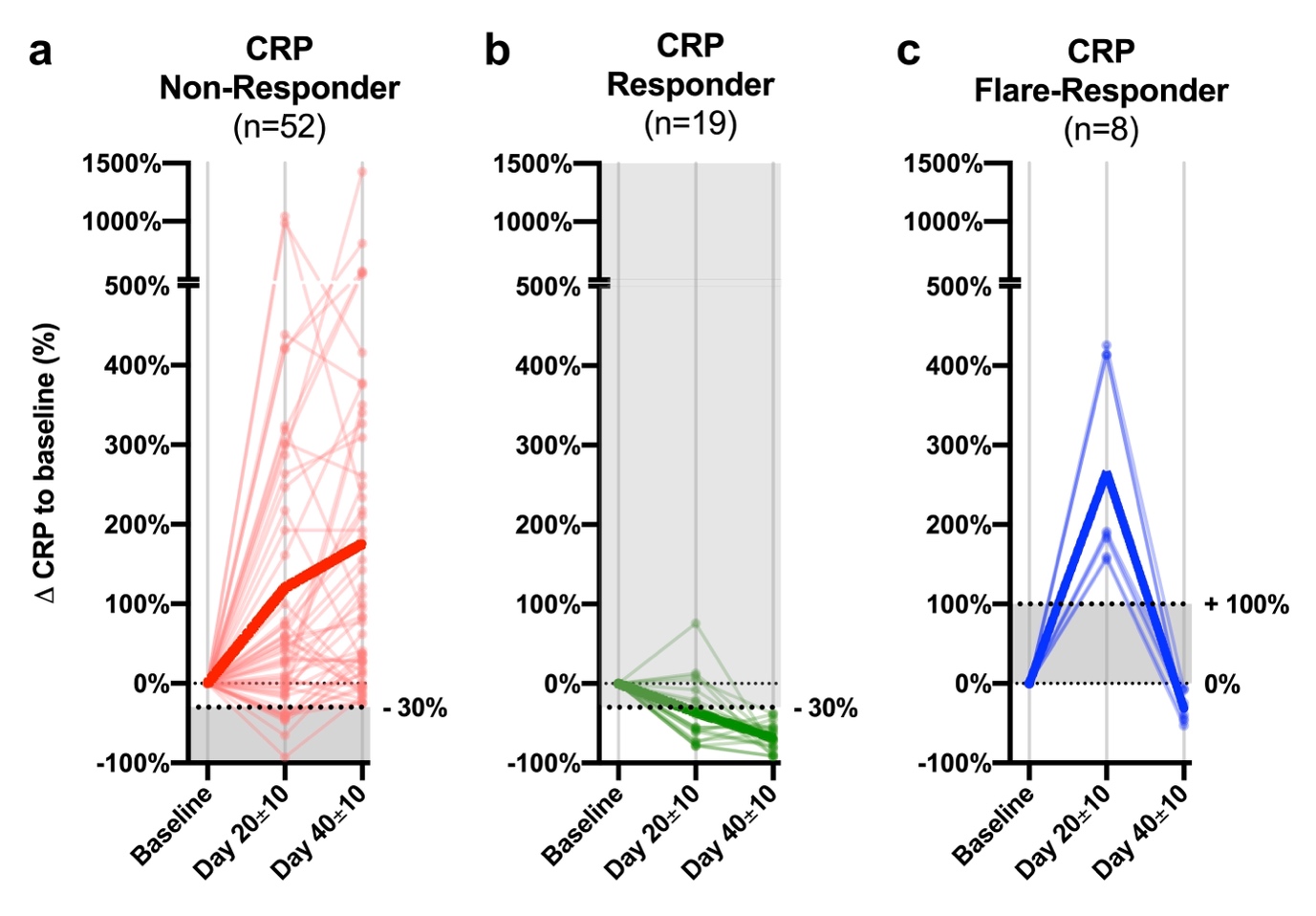
**

**Supplementary Fig. 4** CRP kinetics. Patients were assigned to three groups according to their early CRP kinetics on day 20±10 and day 40±10. CRP responders (b) showed a ≥30% decrease in CRP compared to baseline, CRP flare-responders (c) showed an ≥100% increase of CRP at the first time point and a return to or below baseline by the second time point. CRP non-responders (a) are comprised of the remaining cases. A bold trendline was drawn using segmental linear regression. Individual values are presented as transparent line graphs.

Abbreviations: CRP, C-reactive protein.


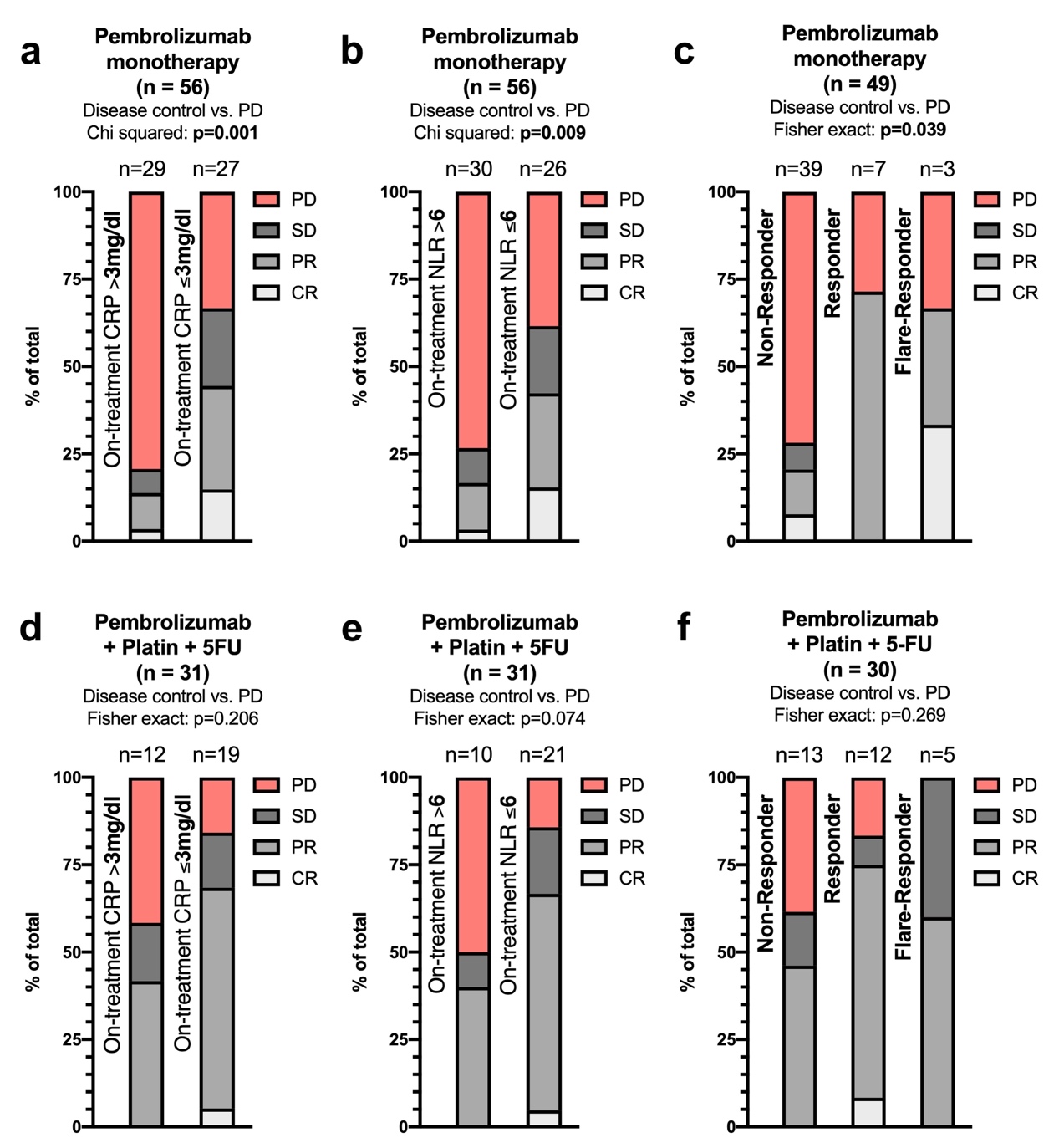


**Supplementary Fig. 5** bOR according to on-treatment CRP, on-treatment NLR and CRP kinetics split by regiment. Pembrolizumab monotherapy (a, b, c) and pembrolizumab with concurrent chemotherapy (d, e, f) is graphed separately. The difference in disease control (SD, PR or CR) between groups was compared using the Chi-squared or Fisher exact test.

Abbreviations: 5-FU, 5-fluorouracil; bOR, best overall response; CR, complete response; CRP, C-reactive protein; NLR, neutrophil-to-lymphocyte ratio; PD, progressive disease; PR, partial response; SD, stable disease.


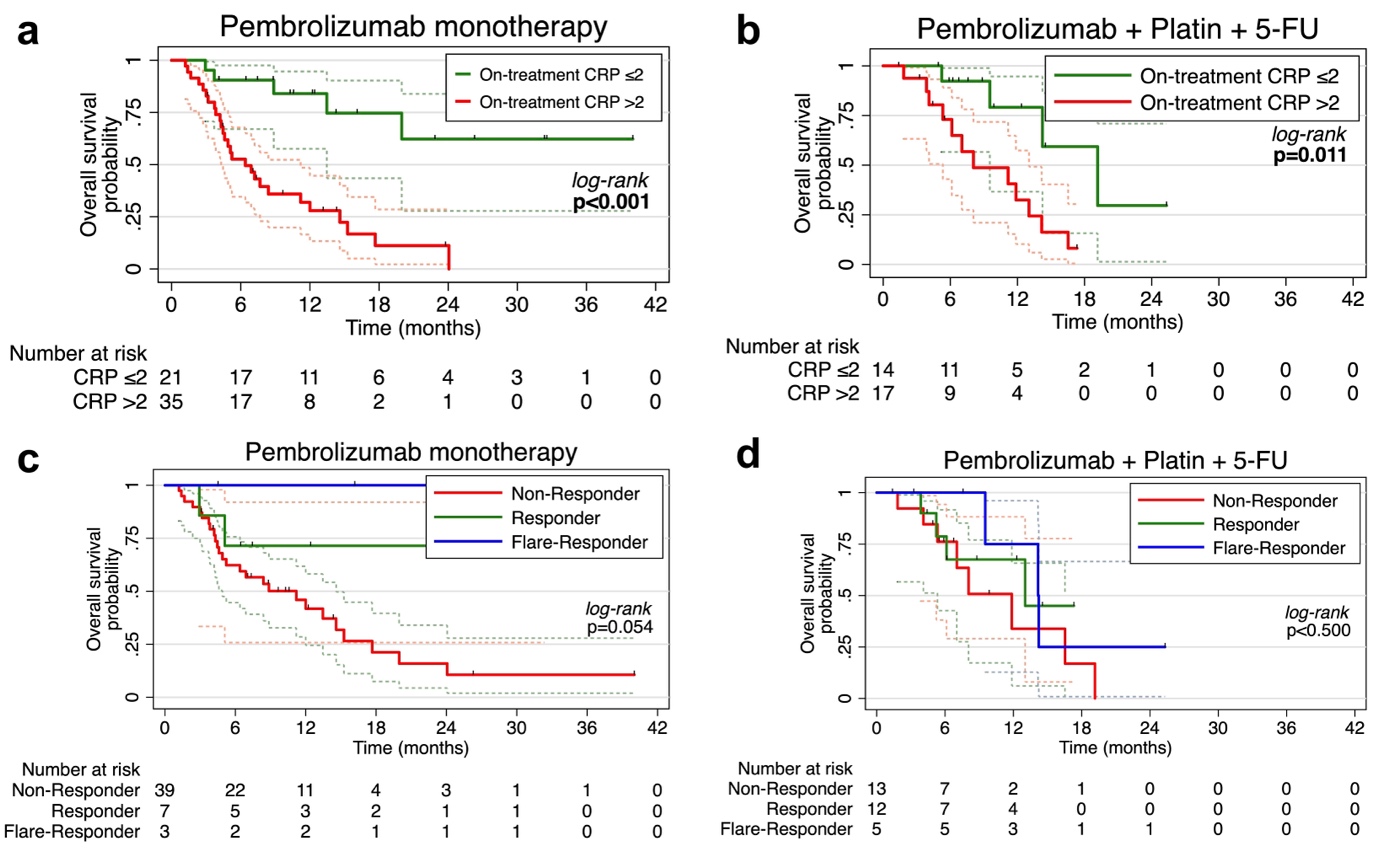


**Supplementary Fig. 6** Survival analysis for OS according to regiment. Kaplan-Meier plots with CI95% (dashed lines) dichotomized by the cross-validated cutoff-points for on-treatment CRP in mg/dl (a, b) levels and CRP kinetics (c, d) are shown separately for pembrolizumab monotherapy and pembrolizumab with concurrent chemotherapy. Log-rank testing was employed for comparison of the survival curves.

Abbreviations: 5-FU, 5-fluorouracil; CI95%, 95% confidence interval; CRP, C-reactive protein; cvAUROC, cross-validated area under the receiver operating curve; NLR, neutrophil-to-lymphocyte ratio; OS, overall survival.


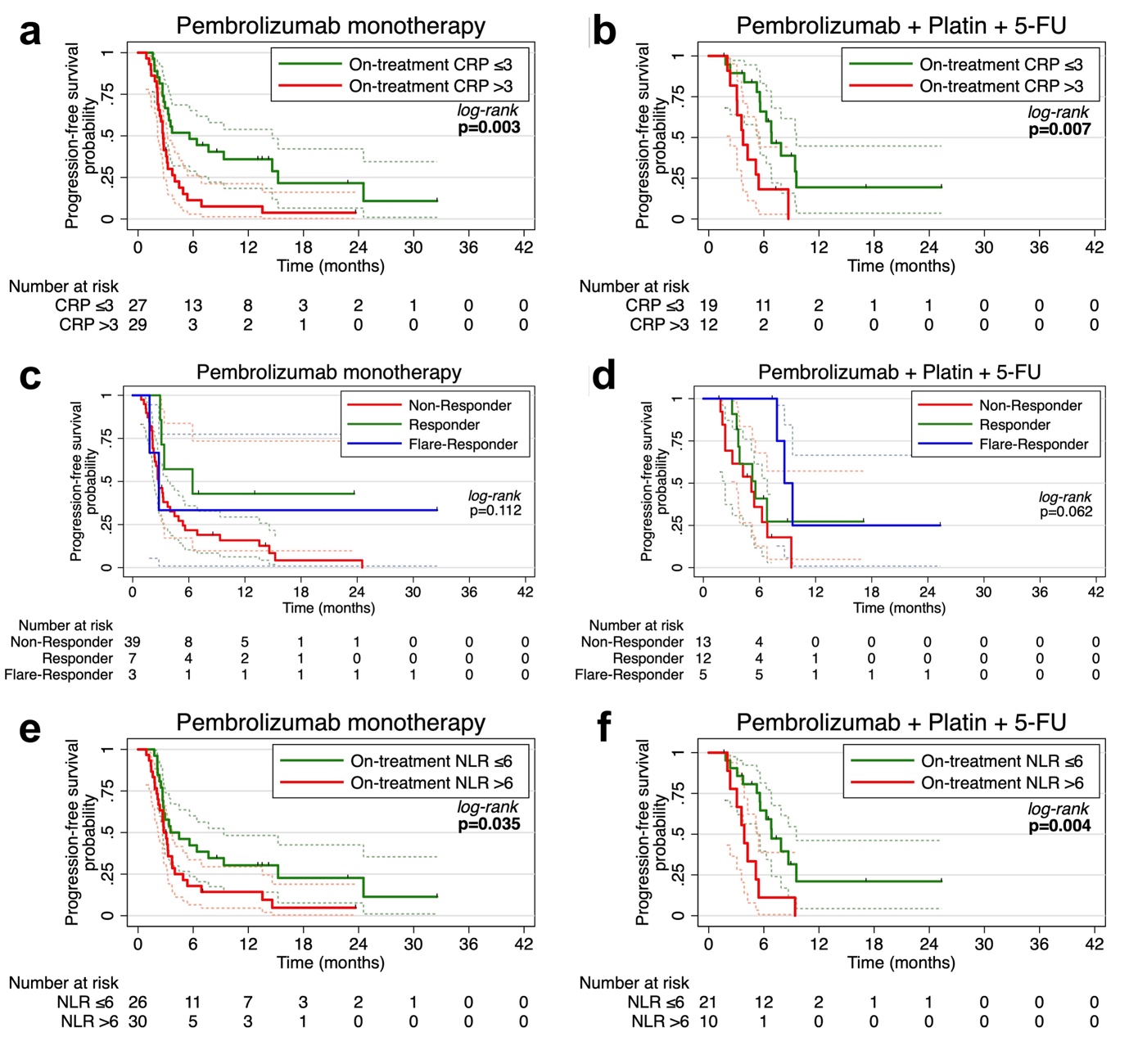


**Supplementary Fig. 7** Survival analysis for PFS according to regiment. Kaplan-Meier plots with CI95% (dashed lines) dichotomized by the cross-validated cutoff-points for on-treatment CRP in mg/dl (a, b) levels, CRP kinetics (c, d) and on-treatment NLR (e, f) are shown separately for pembrolizumab monotherapy and pembrolizumab with concurrent chemotherapy. Log-rank testing was employed for comparison of the survival curves.

Abbreviations: 5-FU, 5-fluorouracil; CI95%, 95% confidence interval; CRP, C-reactive protein; cvAUROC, cross-validated area under the receiver operating curve; NLR, neutrophil-to-lymphocyte ratio; PFS, progression-free survival.
